# Mediastinal Teratoma with Neuroendocrine Features in 34-Year-Old Male with Syncope

**DOI:** 10.1155/2015/153959

**Published:** 2015-12-10

**Authors:** Peter A. Andrawes, Masood A. Shariff, Qing Chang, Fanyi Kong, Frank M. Rosell

**Affiliations:** ^1^Department of Surgery, Staten Island University Hospital, Northwell Health System, 475 Seaview Avenue, Staten Island, NY 10305, USA; ^2^Department of Cardiothoracic Surgery, Staten Island University Hospital, Northwell Health System, 475 Seaview Avenue, Staten Island, NY 10305, USA; ^3^Department of Pathology, Staten Island University Hospital, Northwell Health System, 475 Seaview Avenue, Staten Island, NY 10305, USA

## Abstract

Neuroendocrine tumors that arise in an extragonadal teratoma are extremely rare. Somatic-type malignancy, defined as any sarcoma, carcinoma, leukemia, or lymphoma developing in a germ cell tumor, occurs in approximately 2% of all germ cell tumors. Our case represents a mediastinal mass that was incidentally found in a patient with syncope. Surgical resection confirmed mature teratoma with neuroendocrine features.

## 1. Introduction

Neuroendocrine tumors that arise in an extragonadal teratoma are extremely rare [1]. To the best of our knowledge, very few cases of a neuroendocrine tumor arising in the thoracic cavity as a teratoma have been previously reported [1–3]. Somatic-type malignancy (STM), defined as any sarcoma, carcinoma, leukemia, or lymphoma developing in a germ cell tumor, occurs in approximately 2% of all germ cell tumors. In STM, neuroendocrine carcinoma is extremely rare [1]. In our case, we report a thoracic teratoma with neuroendocrine elements which was found incidentally on thoracic CT-scan of a 34-year-old male patient who presented with multiple episodes of syncope. Malignant transformation in a teratoma is difficult to diagnose on clinical and radiological assessment; histopathology is essential to detect the malignant nature of these neoplasms [3].

## 2. Case Report 

A 34-year-old man with a past medical history of anxiety and hypothyroidism presented to emergency department complaining of nausea, tremors, and syncope. The symptoms had been persistent for the last two weeks. On the day of presentation, the patient had 3 syncopal episodes that occurred while laying down, each lasting for 6 or 7 seconds. The patient denied any history of seizure activity or recent head trauma.

**Figure 1:**
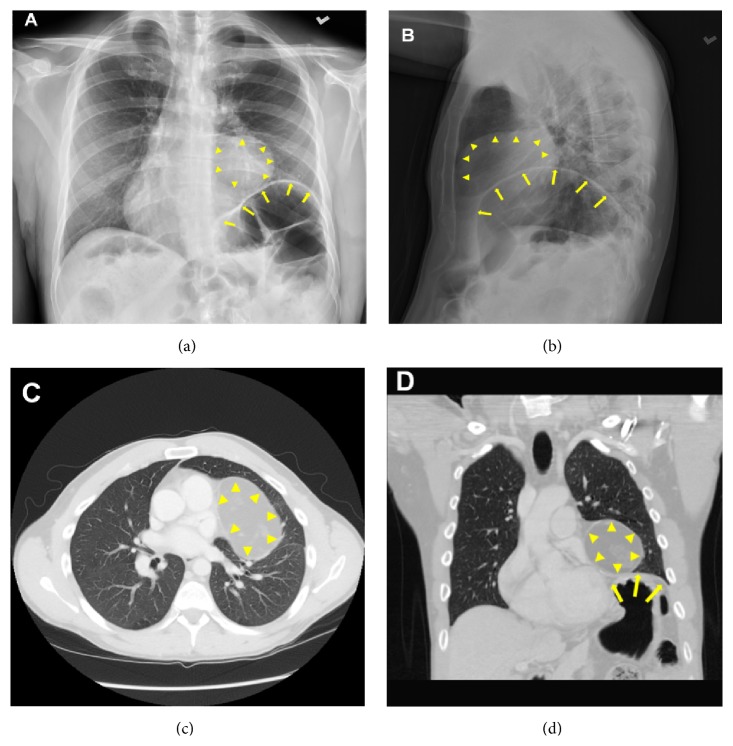
(a) Chest X-ray showing abnormal cardiac figuration with rounded left cardiac border, prominent right cardiac border, and elevated left hemidiaphragm outlines by yellow arrows; rounded mass is seen on top of the raised diaphragm (arrow heads). (b) Chest X-ray-lateral view of the mass (arrow heads) and raised diaphragm (yellow arrows). (c) CAT scan, transverse view of chest showing a well-demarcated heterogeneous, predominantly fat containing mass with a calcified outline, noted arising from the mediastinum (arrow heads), abutting the left cardiac border, and measuring 8.8 × 5.5 × 5.5 cm, with no definite invasion of the cardiac structures; and (d) coronal view of the chest prominently denoting the hemidiaphragm (yellow arrows) and mediastinal mass on top of it (arrow heads).

Physical examinations were unremarkable. Cardiac enzymes and alcohol level were within normal range. Radiology studies with a CT-scan of the head and CT-angiography were unremarkable. Chest X-ray showed abnormal cardiac shadow with rounded left cardiac border, prominent right cardiac border, and elevated left hemidiaphragm (Figures 1(a) and 1(b)). Subsequently, thoracic CT-scan showed a well-demarcated heterogeneous, predominantly fat containing mass arising from the mediastinum, abutting the left cardiac border, and measuring 8.8 × 5.5 × 5.5 cm, without definite invasion of the cardiac structures. Thin peripheral calcification was also noted (Figures 1(c) and 1(d)).

After excluding any cardiac or neurological reasons for his symptoms, the patient was sent for diagnostic chest fluoroscopy, which showed bilateral diaphragms moving symmetrically with no paradoxical motion and with mild elevation of the left hemidiaphragm. Again seen is a 5.8 × 5.4 cm fat containing mass arising from the left side of the mediastinum abutting the left cardiac border. The lungs are clear without focal consolidation or pleural effusion.

**Figure 2:**
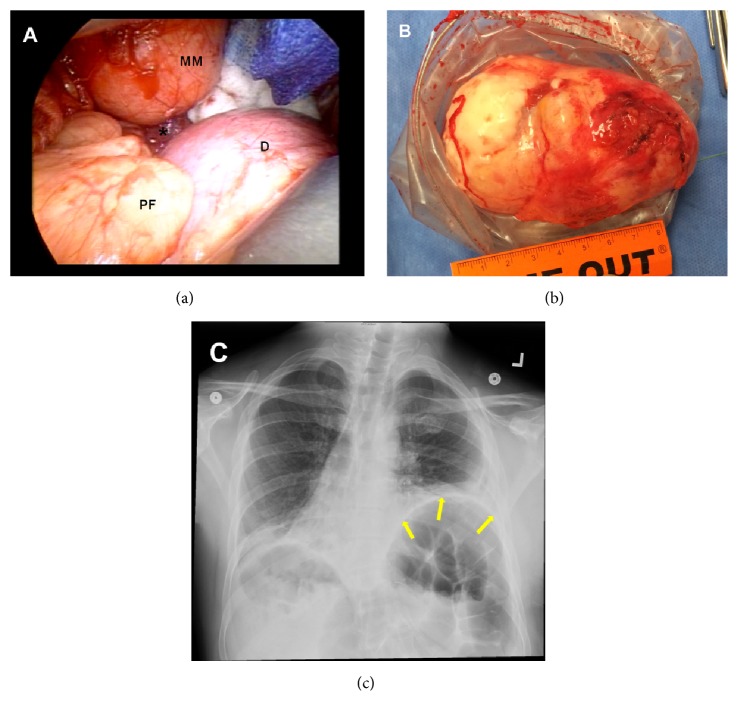
(a) Left thoracotomy/thoracoscopic view of the mass (MM) atop of the diaphragm (D) anterior to the left lung (asterisk) (PF, pericardial fat pad). (b) Gross view of the intact excised mass measuring 10 × 6 × 4.5 cm. (c) Postoperative chest X-ray showing resolution of the left sided mediastinal mass, with no change in the hemidiaphragm (yellow arrows) and the right sided heart.

It was decided to proceed with surgical resection without needle aspiration or biopsy, and the patient underwent left video-assisted thoracoscopic surgery (VATS) and resection of the mediastinal mass. The tumor was found to be adherent to the lateral border of the pericardium, where it was dissected off without resection of the pericardium (Figures 2(a) and 2(b)). The patient tolerated the procedure well and left the operating room with a left chest tube. On postoperative day 1, the chest tube was removed and ambulation was encouraged, with the patient discharged by day 2. Patient's left hemidiaphragm stayed without resolution (Figure 2(c)).

**Figure 3:**
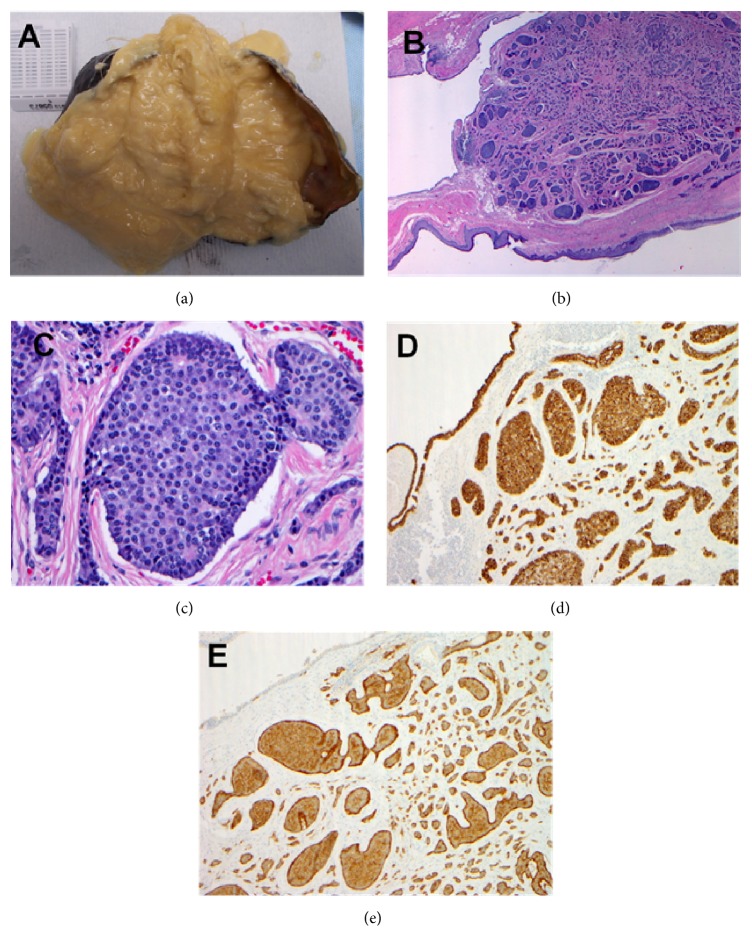
Mediastinal tumor weighed 165 g with smooth outer surface and yellow thick cheese-like material mixed with gray white hair (a). Sectioning of pedunculated nodules (diameter of 0.5 cm) found to have multiple components including skin and respiratory epithelium, which is consistent with a mature cystic teratoma/dermoid cyst (b); well-circumscribed tumor lesions noted with small, normochromatic, uniform tumor cells with an active nucleus (c). The tumor cells were positive for neuroendocrine tumor markers ((d) pancytokeratin stain and (e) chromogranin stain).

Pathologic evaluation weighted the tumor to be 165 g and 10 × 6 × 4.5 cm in size with smooth outer surface. On pathologic dissection, the interior was filled with light yellow thick cheese-like material mixed with gray white hair (Figure 3(a)). There were two indurated thickened areas with smooth inner lining, measuring 3 × 3 × 0.4 cm and 3 × 2.5 × 0.8 cm, respectively. There were two pedunculated polypoid nodules, measuring 0.5 cm in greatest dimension. There was a third area of thickening with filamentous protrusion, measuring 1 × 1 × 0.8 cm. The final pathology showed mature cystic teratoma or dermoid cyst, with inked resection margin-free of tumor. One 0.6 cm nodule of low grade neuroendocrine neoplasm is present in the cystic wall without lymphovascular invasion. The immunohistochemical studies showed positive pancytokeratin, chromogranin, synaptophysin, CD56, and negative P63 markers (Figures 3(b)–3(e)).

## 3. Discussion 

Teratoma is the most common germ cell tumor and usually contains tissue derived from the ectodermal layer, whereas mesodermal and endodermal derived structures are more infrequent [2]. STM is defined as a germ cell tumor accompanied by a nongerm cell tumor such as sarcoma, carcinoma, leukemia, or lymphoma. STM is rare and is observed in ~2-3% of all male germ cell tumors and about one-third occur in the mediastinum [1–5]. In STM, neuroendocrine carcinoma is extremely rare [1]. Mature teratomas represent approximately 60% to 75% of mediastinal germ cell tumors [6, 7].

Patients with a mediastinal tumor can have different presentations. A mediastinal mass is often an incidental diagnosis when patients undergo evaluation for an unrelated condition or symptom like in our case in which the patient presented with nausea and syncope. Clinical symptoms are observed in 62% of patients and include chest pain (30%), dyspnea (16%), fever and chills (20%), and cough (16%) [8]. Of note, tumors with somatic-type malignancy are much more likely to become clinically symptomatic than ordinary germ cell tumors [2, 8]. In some cases, patients present with complaints secondary to local mass effect on adjacent structures, such as respiratory symptoms due to airway compression or swelling due to compression of vascular structures. Other patients developing systemic symptoms that result from the mediastinal mass, such as fever, weight loss, or endocrine syndromes, have been described [6], which lead to discovery of the tumor on subsequent work-up.

A mediastinal mass is typically diagnosed or suspected based upon abnormalities detected accidently on a posterior-anterior and lateral chest X-ray. Findings can vary from subtle mediastinal findings to the obvious presence of a large mass in the mediastinum. A chest CT-scan with intravenous contrast is typically used to evaluate chest X-ray abnormalities. The CT can confirm the presence of a mediastinal mass; occasionally an abnormality that was thought to be a mediastinal mass is found to be something else, such as a diaphragmatic hernia or an aortic aneurysm. It can also provide detailed information on the mediastinal abnormality, including its size, location, relationship to other structures, tissue characteristics, and presence of calcifications, fluid or fat.

Detailed knowledge of any involvement or compression of other mediastinal structures is critical in planning treatment and preparing a patient for possible surgery. Unnecessary morbidity can be avoided if involvement of unresectable structures is identified prior to resection. No imaging beyond the CT-scan is needed in many cases to evaluate the mediastinal mass.

Tumor markers can support the diagnostic work-up in some instances of anterior mediastinal masses when thymoma or germ cell tumor is suspected. Although teratomas comprising areas of immature or neuroendocrine differentiation are generally considered malignant, in localized tumors the prognosis seems favorable particularly in young patients and complete surgical resection is the treatment of choice [2, 6, 9, 10].

The novelty of our case originates from the symptomatic presentation of syncope which initially delineated the differential diagnosis towards neurologic and cardiac causes. The mass effect was not elucidated on history and physical, as patient had long standing history of hypothyroidism and an anxiety disorder. The symptoms and the final diagnosis pointed to a carcinoid syndrome, which could explain the syncope due to an abrupt drop in blood pressure from release of large amounts of hormone or chemical substrate by the teratoma. Although teratomas comprise areas of immature or neuroendocrine differentiated cells, these are generally considered malignant, whereas in localized tumors the prognosis seems favorable particularly in the young and with complete surgical resection confers a positive outlook.
